# Chemical and Antifungal Variability of Several Accessions of *Azadirachta indica* A. Juss. from Six Locations Across the Colombian Caribbean Coast: Identification of Antifungal Azadirone Limonoids

**DOI:** 10.3390/plants8120555

**Published:** 2019-11-29

**Authors:** Juan Manuel Álvarez-Caballero, Ericsson Coy-Barrera

**Affiliations:** 1 Grupo de Química y Bioprospección de Productos Naturales, Universidad del Magdalena, Santa Marta 470004, Colombia; 2 Bioorganic Chemistry Laboratory, Facultad de Ciencias Básicas y Aplicadas, Universidad Militar Nueva Granada, Cajicá 250247, Colombia; ericsson.coy@unimilitar.edu.co

**Keywords:** *Azadirachta indica*, *Fusarium oxysporum*, chemical variability, limonoids, antifungals

## Abstract

Plant materials (i.e., leaves, fruits, and seeds) from 40 trees of *Azadirachta indica* A. Juss. were collected from six different locations across the Colombian Caribbean coast. Eighty-four ethanolic extracts were prepared and the total limonoid contents (TLiC) and the antifungal activity against *Fusarium oxysporum* conidia were measured. Their chemical profiles were also recorded via liquid chromatography-electrospray ionization interface-mass spectrometry (LC-ESI-MS) analysis and the top-ranked features were then annotated after supervised multivariate statistics. Inter-location chemical variability within sample set was assessed by sparse partial least squares discriminant analysis (sPLS-DA) and the chemical profiles and biological activity datasets were integrated through single-*Y* orthogonal partial least squares (OPLS) to identify antifungal bioactives in test extracts. The TLiC and antifungal activity (IC_50_ values) of the *A. indica*-derived extracts were found to be ranging from 4.5 to 48.5 mg limonin equivalent per g dry extract and 0.08–44.8 μg/mL, respectively. The presence/abundance of particular limonoids between collected samples influenced the variability among locations. In addition, the integration of chemical and antifungal activity datasets showed five features as markers probably contributing to the bioactivity, annotated as compounds with an azadirone-like moiety. To validate the information provided by the single-*Y* OPLS model, a high performance liquid chromatography (HPLC)-based microfractionation was then carried out on an active extract. The combined plot of chromatographic profile and microfraction bioactivity also evidenced five signals possessing the highest antifungal activity. The most active limonoid was identified as nimonol **1**. Hence, this untargeted metabolite profiling was considered as a convenient tool for identifying metabolites as inter-location markers as well as antifungals against *Fusarium oxysporum*.

## 1. Introduction

*Azadirachta indica* A. Juss. (called the Neem tree) belongs to the Meliaceae family and has been mainly cultivated and used as a folk medicinal plant in India for thousands of years [1]. However, the presence of this species has been extended to several tropical and subtropical regions worldwide [2]. Almost all parts of this tree have been employed as natural drugs in several parts of Asia, Australia, and Africa [2]. These uses are oriented to various medicinal purposes to treat different chronic and acute human diseases, which are attributed to its capacity to up- or down-regulate multiple cell signaling pathways, involving several targets implicated in cell apoptosis, metastasis, and survival [3]. Additionally, *A. indica* can be also employed as an insecticide, an antibacterial, larvicidal, antiparasiticidal, and antifungal agent [4]. From such a plethora of biological effects, *A. indica* has been the focus of natural product studies, resulting in the isolation of more than 300 compounds from different extracts of this plant [3]. Over 130 of such isolated compounds are limonoid-type metabolites, which are associated with anti-inflammation, anticancer, insecticide, antibacteria, antiprotozoa, and antifungal activities [5]. Limonoids are derived from apotirucallane- or apoeuphane-type triterpenoids when side chains lose four terminal carbons, usually forming a furan ring [6]. Limonoids are divided into various subclasses based on structural characteristics/attributes, which are gathered into four main subgroups such as ring-intact, ring-*seco*, rearranged, and limonoid derivatives [5]. 

While some *A. indica* components have been well-characterized for insect antifeedant effects [7], there is a lack of information regarding antifungal properties of compounds from *A. indica* [4]. However, some investigations have described antifungal extracts/fractions from *A. indica* against several human patrogens [8] and phytopathogens [9,10]. In addition, some isolated compounds such as azadirachtin [11] and other azadirone-type limonoids [12] exhibited antifungal activity against the phytopathogens *Macrophomina phaseolina* and *Puccinia arachidis*, respectively. Regarding *Fusarium oxysporum*, a phytopathogen in Colombia that attacks more than 100 crop plants worldwide [13], seed and leaf extracts of *A. indica* caused growth inhibition of this fungus at different levels [10].

Owing to sustainable pest management, reforestation and soil restoration programs in tropical regions, trees of *Neem* have been planted in several Central and South American countries [14]. In Colombia, *A. indica* has been widely used to restore and improve soil quality in dry lands (e.g., rocky and sandy lands) degraded by overgrazing, salinity, and surface mining, as well as those lands prone to desertification [15,16], especially such zones located along the Colombian Caribbean coast [17]. These practices could promote *A. indica* long/short-term acclimation to counteract the environmental conditions and biotic/abiotic factors of the geographical location as a flexible adaptive response [18]. Such a response is often mediated by the production/accumulation of particular specialized (secondary) metabolites, comprising variability in the metabolite profile for individual accessions growing under diverse environmental conditions [19,20], or the occurrence of particular genetic mutations to adapt the plants to a specific environment, which could also impact metabolite production [20]. The detailed examination of the chemical variations of those plants of the same species from different geographical origins can associate particular metabolites with the location and/or environmental conditions/factors [21]. Such linking is very useful, for instance, towards the quality control of botanical and food products [22]. This chemical variation can be also related with the respective biological activity dataset (i.e., each plant product from each accession is separately evaluated for a certain biological activity) to discriminate bioactives in the most active extracts. The integration of chemical and environmental/biological activity datasets can be achieved using untargeted/targeted metabolomics for detecting/identifying markers under supervised statistical analysis, which is being broadly employed for its advantages [21,23]. So far, such an integration has not been carried out for *A. indica* in any previous study, although a work evaluated the variation of azadirachtin from different trees and/or origin [24]. 

Thus, in the current course of our research on antifungals of plant origin against *F. oxysporum*, we studied the chemical and antifungal activity variation of several extracts from different accessions of *A. indica*, which were collected from six different locations across the Colombian Caribbean coast. In this regard, the liquid chromatography-mass spectrometry (LC-MS)-based chemical profiles were integrated with locations and antifungal activity as a strategy to identify makers in the set of different extracts of *A. indica* trees. The present work constitutes the first study on characterizing the chemical and antifungal variation of different *A. indica* accessions.

## 2. Results and Discussion

**Figure 1:**
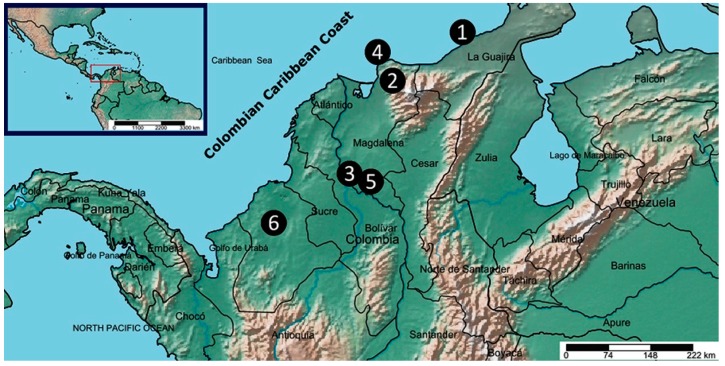
Geographical distribution map of collected *Azadirachta indica* samples. Circled numbers are related to the six different collecting locations: (**1**) = Riohacha (R), (**2**) = San Pedro de la Sierra (SPS), (**3**) = Santa Ana (SA), (**4**) = Santa Marta (SM), (**5**) = Mompox (M), and (**6**) = Montería (Mo). Map prepared/adapted using the online tool SimpleMappr (http://www.simplemappr.net).

Six different locations were selected to collect randomly distinct accessions of *A. indica* from the Colombian Caribbean coast, as shown in Figure 1. This Colombian region is administratively comprised by the departments of Atlántico, Bolívar, Cesar, Córdoba, La Guajira, Magdalena, and Sucre. The region is located to the north of the country, occupying a geographical area of 132,244 km^2^ and characterized by arid, coastal mountain, flood, and plain areas [25]. Therefore, locations were then chosen according to this topography diversity of the Caribbean coast, covering the main relief areas such as La Guajira Peninsula, The Sierra Nevada de Santa Marta, the Magdalena River, and Cienaga Grande and Sinú Belt (Table 1) [25]. According to the Köppen–Geiger climate classification system, the six locations are related to hot semi-arid (BsH), tropical monsoon (Am), and tropical wet (savanna) (Aw) climatic tropical zones. They have annual mean temperatures of 18.9–28.3 °C, an annual mean rainfall range of 512–2721 mm, and altitudes between 5 and 1378 m above sea level (Table 1). Having the locations topographically identified, also depending on the tree abundance of *A. indica* species (*n* > 5), plant parts of each accession were therefore sampled depending on the natural offer of the tree, between July to August, 2018, a dry season in this region. In Table 1 is also listed the information regarding collecting locations (i.e., zones and climate conditions), number of accessions and plant parts. A total of 40 trees were sampled and 84 plant samples were then collected. The coordinates for each accession are registered in Table A1. 

### 2.1. Total Limonoid Contents and Antifungal Activity of A. indica-Derived Extracts

Once the respective plant materials were appropriately sampled (Table 1), the preliminary study on the characterization of limonoid content and the antifungal activity was started. Thus, plant materials (i.e., leaves, fruits, and seeds) were initially lyophilized and extracted with 96% ethanol to afford crude ethanolic extracts. A total of 84 raw extracts were prepared from those samples per location registered in Table 1. To remove fatty components, a portion of each dried crude extracts (50 mg) was re-dissolved in methanol to perform a C18 solid-phase extraction (SPE) pre-treatment step. The resulting amount of each SPE extract was then divided into two subsamples in order to measure the total limonoid content (TLiC), expressed as mg limonin equivalents per gram dry extract (mg limonin/g DE), on one hand, and the inhibition of *F. oxysporum* conidia germination, expressed as half maximal inhibitory concentration (IC_50_ in μg/mL), on the other. Table A2 and Table A3 contain the results of TLiC and antifungal activity of the resulting eighty-four extracts of *A. indica*. 

**Figure 2:**
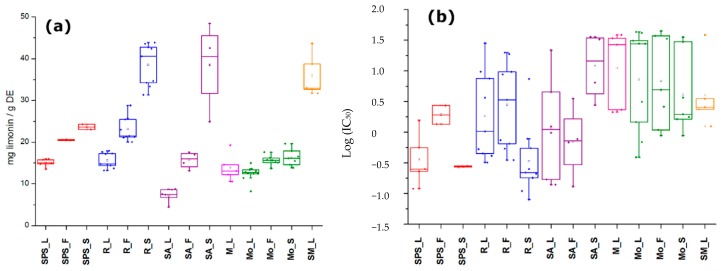
Boxplot for the distribution of (**a**) total limonoid contents (expressed as mg limonin/g dry extract (DE)) dataset and (**b**) antifungal activity as IC_50_ values among extracts from the same location (SPS = San Pedro de la Sierra; R = Riohacha; SA = Santa Ana; M = Mompox; Mo = Monteria; SM = Santa Marta) and plant part (L = leaves; F = fruits; and S = seeds).

Regarding TLiC, extracts exhibited contents ranging from 4.5 to 48.5 mg limonin/g DE (Table A1). A seed sample from an accession collected in Santa Ana (SA) exhibited the highest TLiC value, while a sample of leaves from the same accession in SA showed the lowest. Seeds generally exhibited the highest limonoid amounts in comparison to the other plant parts of each location, which is in agreement with previous studies using the vanillin assay for colorimetric limonoid quantification [26]. In contrast, leaves exhibited the lowest mean TLiC values among sample set but these values were higher to that of other reported work [27]. On comparing the mean values per location and per plant part (Figure 2a), seed extracts from SA and Riohacha (R) exhibited similar values and no significant differences (*p* > 0.05) were found between the seeds from accessions of these two locations. However, some outliers appeared in each sample set. In general, samples from San Pedro de la Sierra (SPS), R and SA exhibited significant differences (*p* < 0.05) in the mean TLiC values among plant parts, while samples from Montería (Mo) displayed similar TLiC values between plant parts and similar to that of Mompox (M). Leaves from Santa Marta (SM) showed higher TLiC values, similar to those of seed samples from SA and R. In general, regarding the location characteristics, no correlation was found between TLiC and climatic factors. Owing to the variations among accessions of a particular location, the limonoid content variation could be affected by other abiotic/biotic factors, such as soil type or ecological environment [18]. Consequently, it was previously stated that climatic conditions such as humidity, rainfall, and temperature did not affect the azadirachtin content in several neem trees from different agroclimatic regions of India [24].

The antifungal activity (in terms of IC_50_ values) of *A. indica* extracts against *F. oxysporum* conidia were found to be ranged between 0.08 and 44.8 μg/mL (Table A2). The comparison of the mean logarithmic IC_50_ values per location and per plant part (Figure 2b) disclosed that the antifungal activity exhibited a high dispersion among plant parts of accessions from the same location, excepting seed extracts from SPS (i.e., IC_50_ = 0.28 and 0.27 μg/mL). Some seed samples from R showed the strongest antifungal activity and certain leaf samples from Mo were the weakest ones. However, leaf samples from SPS also exhibited strong antifungal activity (IC_50_ < 1.56 μg/mL). In that sense, it was reported that *A. indica* extracts from seeds and leaves caused growth inhibition of *F. oxysporum,* being this phytopathogen the most sensitive one among other tested fungi [10]. No relationship was found between TLiC and IC_50_ values (Pearson correlation coefficient <−0.2).

### 2.2. Chemical Variability through Metabolic Profiling of Crude Extracts of A. indica

**Figure 3:**
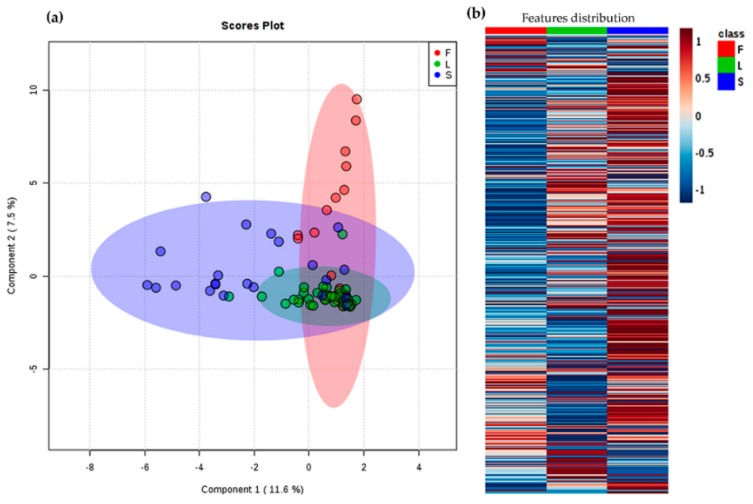
Differentiation of *A. indica* samples under the supervision of plant part (L = leaves; F = fruits; and S = seeds); (**a**) Principal component 1 (PC1) vs. PC2 score plot by sparse partial least squares discriminant analysis (sPLS-DA). Total explained variance of each component is shown in the axes of the score plot. (**b**) Heat map on abundances of all detected *m/z* features. Each colored cell on the map corresponds to the abundance for each feature (dark blue = low; dark red = high).

The chemical variability among extracts was also studied through metabolic profiling in order to expand the chemical characterization of *A. indica* accessions beyond total limonoid content. Thus, liquid chromatography-electrospray ionization interface-mass spectrometry (LC-ESI-MS)-based profiles were recorded for each plant extract (*n* = 84) and the *m/z* features were retrieved from the raw profiles, under pre-processing with MZmine 2.2 [28]. A total of 561 features (i.e., metabolites) and 84 observations (samples) were compiled into a data matrix. This matrix was employed to observe the chemical variation under supervised statistics using categorical variables (i.e., classes) by sparse partial least squares discriminant analysis (sPLS-DA). The first analysis was supervised by the plant part (i.e., leaves (L), fruits (F), and seeds (S)) as categorical variable, regardless of the location factor. The resulting score plot is presented in Figure 3a. Leaf extracts exhibited the lowest variability in comparison to those of fruits and seeds, but clear clusters cannot be clearly evidenced. An intuitive visualization of the mean abundance distribution of *m/z* features for each plant part dataset, as presented in the heatmap in Figure 3b, can complement and support such a clustering. On comparing the three plant parts, the most contrasting patterns can be observed between fruits and seeds, since seed samples exhibited higher number of the most abundant features while fruits showed a lower number. However, the total explained variance of this sPLS-DA model was found to be very low (19.1%), indicating the discrimination is not reliable, due to high variations among extracts from different locations. In this way, an analysis using location as supervision variable between plant parts was then required. 

**Figure 4:**
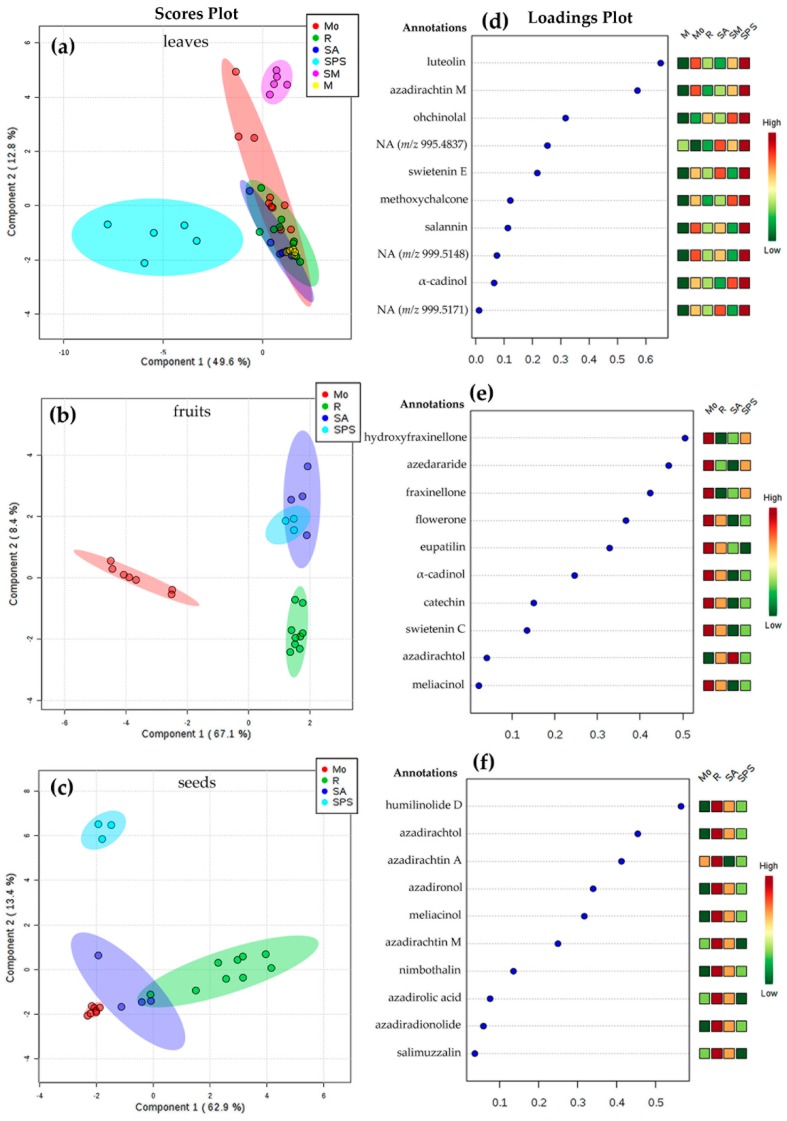
Sparse partial least squares discriminant analysis (sPLS-DA)-based differentiation of *A. indica* samples under the supervision of location (SPS = San Pedro de la Sierra; R = Riohacha; SA = Santa Ana; M = Mompox; Mo = Monteria; and SM = Santa Marta); Score plot by sPLS-DA for (**a**) leaves, (**b**) fruits, and (**c**) seeds. Loadings plot from sPLS-DA model for the PC1, top-ranking the selected *m/z* features for (**d**) leaves, (**e**) fruits, and (**f**) seeds. Total explained variance of each component is shown in the axes of each score plot. Selected features were annotated (level 3) using chromatographic and UV and MS spectral data and following the parameters of the Metabolomics Standards Initiative (MSI) [29]. NA = not annotated.

Therefore, a second analysis was performed, dividing the main dataset into three subdatasets related to the plant parts. Hence, each subdataset was separately analyzed by sPLS-DA using location as supervision variable. The resulting score plots are presented in Figure 4a–c. 

Regarding the chemical variation of leaves extracts, SPS and SM locations were discriminated in different groups, which can be rationalized by the different climatic conditions, especially for SPS (Table 1). Mo location exhibited the highest dispersion, whereas leaves extracts from R, SA, and M were found to have similar metabolic profiles (Figure 4a), which could not be related to the climatic conditions. In the case of fruit and seed extracts, the chemical variations could not be also related to the climatic conditions. However, the groups were clearly clustered regarding location, excepting fruits from SA and SPS, which appeared to comprise similar profiles (Figure 4b). Fruits from Mo and seeds from SPS were mostly different from other locations. The total explained variance of this sPLS-DA models (62.8%–76.3% range) are sufficient to discriminate *A. indica* samples of different plant parts and locations used in the present study [30], but other biotic/abiotic factors, in addition to climatic conditions and genetics, should be included to rationalize such variations in further studies [18,19].

Figure 4d–f exhibited the sPLS-DA loadings plots along principal component 1 (PC1) top-ranking the most influencing features for the discrimination of plant part samples among different locations. PC2 was not considered due to the low explained variance (<12%). These selected features were then annotated (identification at level 3 [29]) using the chromatographic and mass spectral (MS) data. The respective results are presented in Table A4. SPS location exhibited the highest relationship of top-ranked features from leaves, characterized by the presence of flavonoids, limonoids, terpenes, and three unannotated features. In contrast, R location was highly correlated to selected features from seeds, corresponding to limonoid-like metabolites. In the case of fruits, ranked features exhibited an association to Mo location, by the presence/abundance of limonoids, limonoid derivatives, terpenes and flavonoids, excepting fruits from SA, which were influenced by one feature annotated as azadirachtol.

### 2.3. Integration of Chemical and Antifungal Activity Datasets for Detecting Bioactives

**Figure 5:**
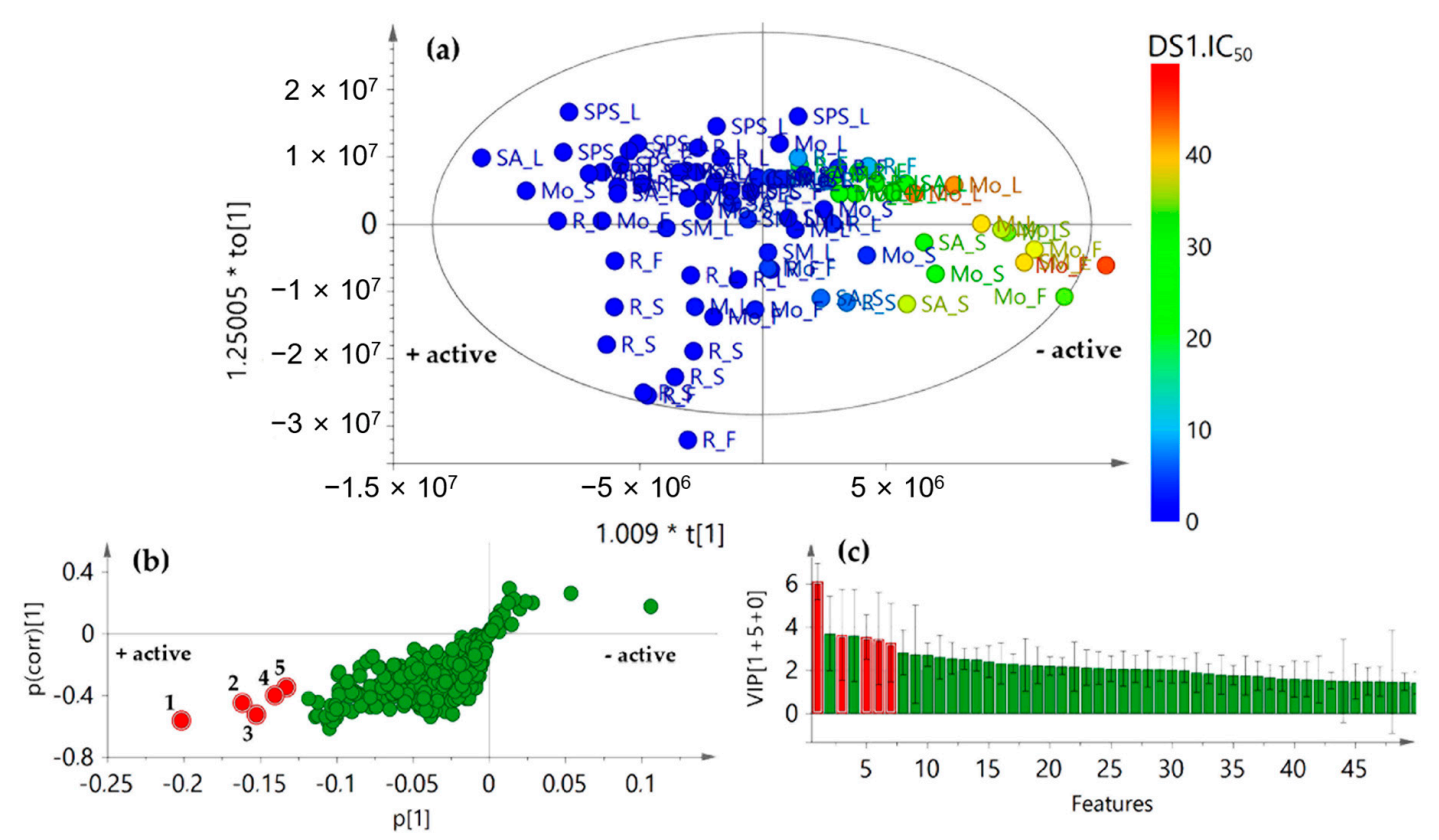
Single-*Y* orthogonal partial least squares (OPLS) model [2+2+0] after integration of chemical and biological activity datasets to identify bioactives. (**a**) Score plot of chemical datasets (*X*) supervising with antifungal activity (*Y*) as IC_50_ values (R^2^*X*_(cum)_ = 0.728, R^2^*Y*_(cum)_ = 0.779, and Q^2^_(cum)_ = 0.720); (**b**) *S*-plot from Single-*Y* OPLS, highlighting in red circles the most influencing features **1**–**5**; (**c**) Variable importance for the projection (VIP) plot (95% jack-knifed confidence intervals), highlighting in red circles the most influencing features (**1**–**5**).

Information regarding extract composition (i.e., TLiC) and antifungal activity separately did not reveal a clear association with presence/absence of bioactive compounds in test extracts. Thus, the integration of biological and chemical data, such as the inhibition of conidia germination (dependent) with MS-derived feature (independent), was employed as a tool for identifying antifungal components. Therefore, a biochemometrics-based analysis through the internal cross-validated construction of a single-*Y* orthogonal partial least squares (OPLS) model was then performed. The respective score plot (Figure 5a) evidenced some correlation based on bioactivity (R^2^*X*_(cum)_ = 0.728, R^2^*Y*_(cum)_ = 0.779, and Q^2^_(cum)_ = 0.720), since the most active extracts were clustered oppositely to the less active ones along principal component 1. This fact rationalized an existing association between antifungal activity and phytoconstituents in test extracts. 

In order to recognize such constituents, the analysis was expanded to visualize the correlation structure and covariance between the X-variables and the predictive score t, using the OPLS-derived *S*-plot. Consequently, the resulting *S*-plot displayed graphically the influence of loadings (i.e., features) against the dependent variable. Therefore, each point (i.e., Rt-Exact mass) in *S*-plot corresponds to a marker ion. For this biochemometrics-based model, the negative, lower quadrant of the *S*-plot contributed the most to statistically differentiating more versus less active marker ions. Thus, five markers (**1**–**5**) were recognized (highlighted as red circles) to possibly have the highest contribution to the observed inhibition of conidia germination (Figure 5b). Marker ion **1** (i.e., compound **1**) exhibited the highest correlation value in *S*-plot according to the variable importance for the projection (VIP) plot using 95% jack-knifed confidence intervals (Figure 5c).

The recognized features **1**–**5** were also annotated after detailed analysis of the chromatographic and MS data [31] (Table 2), but they were found to be different from those top-ranked in sPLS-DA-derived loading plots after integrating locations and chemical data. Hence, metabolite variations according to growing places could be considered different to those related to antifungal activity in these *A. indica* specimens. This fact is in agreement with the environment-driven plasticity of intraspecific metabolite patterns due to ecological pressures and trait variations [32].

The annotated compounds were found to be related to azadirone-like limonoids from the detailed examination of quasi-molecular ions and some detected fragment ions (such as *m/z* 95.0489, 135.0812, and 165.1061) in comparison to the information reported in a previous high-throughput screening [31]. Thus, compounds **1** and **5** were annotated as isomers of hydroxyazadirone, while compounds **2** and **3** were annotated as epoxy and dehydrohydroxyazadirone, respectively. This class of tetranortriterpenes and derivatives have exhibited potent in vitro cytotoxic activity against several cancer cell lines [5,33] and other biological activities [34]. However, there is a lack of information about the antifungal activity of this kind of limonoids against phytopathogens. A previous study reported that nimonol **1** and isomeldenin **3** were active against the fungus *Puccinia arachidis* Speg., the causal agent of the groundnut rust disease, exhibiting a 80% reduction of the pustules per leaflet on day 13 [12].

### 2.4. Validation of the OPLS Model by Direct Identification of Bioactives after High Performance Liquid Chromatography (HPLC)-Based Microfractionation

**Figure 6:**
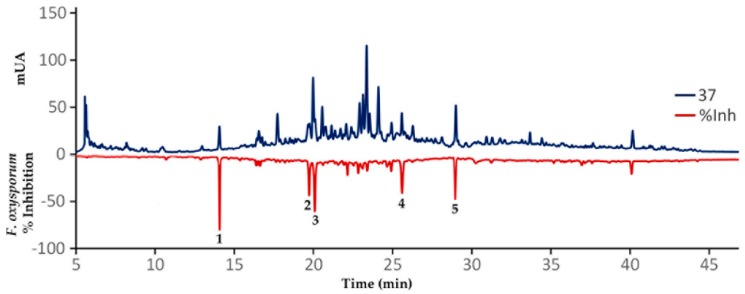
Resulting biochromatogram after microfractionation using an analytical-scale high performance liquid chromatography (HPLC) in comparison of the effect of each microfraction on the viability of *F. oxysporum* conidia. Dark blue line = HPLC profile of extract 37 at 270 nm; Dark red line = % inhibition of *F. oxysporum* conidia germination.

Annotated compounds **1**–**5** can be accordingly considered as promising antifungals. However, a further experimental validation of these findings, after integrating the chemical and antifungal activity datasets by OPLS analysis, was therefore required. Hence, a reversed-phase analytical-scale high performance liquid chromatography (HPLC) separation, involving a time-based microfractionation (slicing each 0.25 min), was then conducted using the extract #37 (a leaf extract from SA, accession 1, code SA_L_1, Table A3). This extract was selected by the measured antifungal activity (IC_50_ = 0.14 μg/mL) as well as the available amount as a rapid strategy to identify bioactives in this extract and drive their isolation within a chromatographic profile [35]. The separation comprised a 50 min-gradient elution method at 270 nm, aiming to separate as many metabolites as possible directly from the selected extract, which were previously treated with C-18 solid phase extraction (SPE) cartridges (500 mg, 6 mL). This SPE-depurated extract #37 (15 mg/mL in MeOH) was separated after 5 μL-injections and the microfractionation procedure was repeated three times. As consequence, 168 microfractions (within the 5 to 47 min range) were independently collected into 3.5 mL tubes. Each resulting microfraction was separately assessed for the in vitro inhibition of *F. oxysporum* conidia germination. The antifungal activity of each microfraction was plotted according to the respective retention time to afford the resulting biochromatogram as presented in Figure 6. Recently, a high-resolution profiling was used to identify several bacterial growth inhibitors in extracts from water and ethanol extracts of 88 traditionally-used plant species [36].

As predicted by the OPLS model, five HPLC peaks were related to the most active microfractions in the range from 45% to 82% inhibition of *F. oxysporum* conidia germination (Figure 6). Other microfractions exhibited antifungal activity (>10% inhibition). The nuclear magnetic resonance (NMR) analysis of the most active microfractions, which were purified by a further semipreparative-scale HPLC separation, indicated that these peaks were precisely related to the compounds **1**–**5**, previously annotated as shown in Table 2, in comparison with the available data for limonoids [37]. The ^13^C NMR profiles were very similar between compounds **1**–**5** with specific differences in *δ_C_* chemical shifts. Thus, the isomeric relationship of compounds **1** and **5** were found to be related to the contrasting position of hydroxyl and acetyl groups at C-6 and C-7 (*δ_C_* 68.2 (C-6) and 79.0 (C-7) in **1** versus 76.4 (C-6) and 68.4 (C-7) in **5**). The epoxy group was located in C-14 and C-15 in compound **2** (*δ_C_* 73.0 (C-14) and 57.3 (C-15)), while a 1,2-dehydro moiety (*δ_C_* 33.5 (C-1) and 38.3 (C-2)) and 16-keto and 1-oxobutan-2-yl moieties (*δ_C_* 208.0 (C-16) and 203.1 (C-21)) were the structural features of compounds **3** and **4**, respectively. 

**Figure 7:**
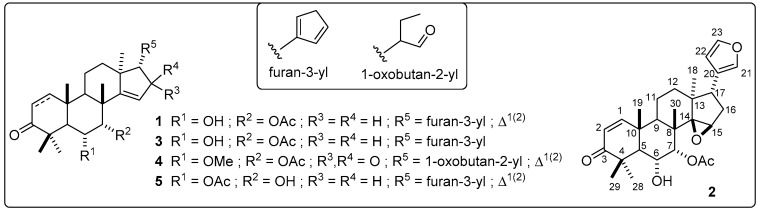
Structures of the identified, most-active azadirone-like limonoids.

In this regard, the high-resolution MS (HRMS) and ^1^H and ^13^C NMR analyses led to the direct identification (at level 1) of the five known azaridone-like limonoids such as nimonol (**1**) [38], 14,15-epoxynimonol (**2**) [39], isomeldenin (**3**) [38], zafaral (**4**) [40], and *O*-acetyl-7-deacetylnimocinol (**5**) [41]. ^1^H and ^13^C NMR data of identified limonoids **1**–**5** are organized in Table A5, whose structures are presented in Figure 7. These findings therefore confirm the usefulness of the biochemometrics approach for azadirone-like antifungals identification from *A. indica* extracts.

The IC_50_ values for the five purified azadirone-like limonoids are presented in Table 3. Limonoid **1** exhibited the most potent antifungal effect (1.48 ± 0.11 μM), while compound **3** showed the weakest activity (15.17 ± 0.22 μM). The presence of a 14,15-epoxy group and the loss of the 1,2-unsaturation led to a diminished inhibition of conidia germination. Similarly, the opposite location of substitutions in C-6 and C-7 involved an almost six-fold reduction of antifungal activity. 

## 3. Materials and Methods

### 3.1. Plant Collection

Plant materials (leaves, seeds, and fruits) of forty *A. indica* trees were obtained from six different locations across Colombian Caribbean coast (Figure 1) between July to August, 2018. Details of such locations are listed in Table 1, related to coordinates, altitude, and climate characteristics (according to the Köppen–Geiger climate classification system, comprising hot semi-arid (BsH), tropical monsoon (Am), and tropical wet (savanna) (Aw) tropical zones). Collecting locations were selected according to the *A. indica* tree abundance (*n* > 5) and plant parts of each accession were then sampled depending on the natural offer of the tree at the moment of sampling. A total of eighty-four plant samples were then stored. Specimens were identified by botanist Eduino Carbonó. Vouchers of collected plant materials were deposited in the Herbarium of the University of Magdalena (UTMC). 

### 3.2. Preparation of Crude Ethanolic Extracts

Plant materials (100 g) were milled under liquid nitrogen and lyophilized using a Scientz 18N freeze dryer (Ningbo Scientz Biotechnology Co., Ningbo, China). Dried plant materials were then extracted with 96% ethanol and stainless steel beads using a MaxiMix™ II Vortex (Thermo Fisher Scientific, Waltham, MA, USA), during 30 min, to afford eighty-four crude ethanolic extracts. A portion of each dried, crude extracts (50 mg) was re-dissolved in methanol and a solid-phase extraction (SPE) pre-treatment step was performed using Strata® C18-U cartridges (55 µm, 70 Å, 100 mg, 1 mL) (Phenomenex, Torrance, CA, USA) to remove fatty components, especially in seeds and fruits. Each SPE-treated extract was then divided into two subgroups (10 mg) to measure the total limonoid content (TLiC) and the inhibition of *F. oxysporum* conidia germination. All extracts were stored at −20 °C until chemical and antifungal analyses.

### 3.3. Measurement of Total Limonoid Contents (TLiC) 

Total limonoid contents (TLiC) of each SPE extract was measured following the colorimetric method described previously with slight modifications [42]. Briefly, extracts solutions (5 mg/mL) were separately disposed in wells (10–40 µL) of 96-well plates in four replicates. Subsequently, a 4-(dimethylamino)benzaldehyde (4-DMAB) (Sigma, St. Louis, MO, USA) solution (100 µL, 37 mg/mL in stock acid solution) and a stock acid (70% perchloric acid and glacial acetic acid 4:5) solution (100 µL) were then added to each well. The resulting mixtures were allowed to react for 40 min at room temperature and under darkness. After incubation time, the absorbance at 500 nm was then measured using a Varioskan^TM^ LUX 96-well plate reader (Thermo Fisher Scientific, Waltham, MA, USA). A calibration curve (absorbance versus limonin in µg/mL) was previously constructed to express the measured values as TLiC as milligrams of limonin equivalents per g of dry extract (DE).

### 3.4. Analysis of the Effect of A. indica Extracts on Viability and Germination of F. oxysporum conidia

*F. oxysporum* strain (LQB-03) was retrieved from infected plants of Cape gooseberry (*Physalis peruviana*) in Cajicá, Colombia. This strain has been preserved on Whatman paper # 1 at −20 °C, and reactivated in potato dextrose agar (PDA) at full concentration before use. Effect of *A. indica* extracts (*n* = 84) on conidia viability was tested by 3-(4, 5-dimethyl-2-thiazolyl)-2, 5-diphenyl-2*H*-tetrazolium bromide (MTT) (Sigma-Aldrich, Waltham, MA, USA) assay [43]. Briefly, freshly prepared *F. oxysporum* conidia suspension (1 × 10^6^ conidia/mL) was cultured in potato dextrose broth (PDB) (200 μL) and exposed to different concentrations of extracts (100 µL, 0.1–100.0 μg/mL in PDB with 2% dimethyl sulfoxide (DMSO) in 3.5 mL test tubes. Treated and control cultures were maintained at 25 °C and 120 rpm for 20 h in a IST-3075 incubated shaker (Lab Companion, Seoul, Korea). Then, culture was centrifuged at 4000 rpm for 15 min and conidia were collected and washed with PBS. For the assessment of viability, control and extract-treated conidia were allowed to react with 100 μL of MTT solution (2 mg/mL) for 16 h on an incubated shaker (160 rpm at 20 °C). Tubes were centrifuged (10,000 rpm, for 5 min), the medium was removed and 1-propanol (500 µL) was added to each one. Tubes were finally vortexed, centrifuged (10,000 rpm, for 5 min) and the supernatant (150 µL) of each tube was transferred into wells of a 96-well plate. Conidia viability was evaluated by measuring the optical density (OD) at 560 nm using a Varioskan^TM^ LUX 96-well plate reader (Thermo Fisher Scientific, Waltham, MA, USA). Each treatment consisted of four analytical replicates. Inhibition percentage of each treated conidia was calculated using the OD value of the control (unexposed conidia) taken as 100% viability and related to the OD of each treatment. The half maximal inhibitory concentrations (IC_50_ in μg/mL) for each extract were calculated through non-linear regression from the dose-response curves using the software GraphPad Prism 5.0 (GraphPad software, San Diego, CA, USA). 

### 3.5. Liquid Chromatography Coupled to Mass Spectrometry (LC-MS) Analysis and Peak Annotations

All SPE-treated extracts were analyzed using reverse phase ultra-high performance liquid chromatography (UHPLC) coupled to a diode array detector (DAD) on a prominence Ultra-Fast Liquid Chromatographic (UFLC) system (Shimadzu, Columbia, MD, USA) and a micrOTOF-Q II mass spectrometer (Bruker, Billerica, MA, USA). A Kinetex^®^ column (Phenomenex, Torrance, CA, USA) (150 × 4.6 mm, 2.6 µm) was used for analysis at 0.7 mL/min with a combination of solvent A (1% formic acid in acetonitrile (ACN)) and solvent B (1% formic acid in H_2_O). A gradient elution method was used: (0–1 min 0% B, 1–10 min 0% to 50% B, 10–11 min 50% B, 11–15 min 50% to 100% B, 15–17 min 100%, and 17–19 min 100% to 0% B). The monitoring wavelength was 270 nm. Electrospray ionization interface (ESI) was operated in a positive ion mode (scan 100–2000 *m/z*), desolvation line temperature at 250 °C, nitrogen as nebulizer gas at 1.5 L/min, drying gas at 8 L/min, quadrupole energy at 7.0 eV, and collision energy 14 eV. Peak annotations were performed after detailed scrutiny of the ultraviolet-visible (UV-Vis) and high-resolution mass spectral (exact mass of quasi-molecular and fragments ions) data of each signal in comparison to the information previously reported in literature to Azadirachta species [31,44,45].

### 3.6. Microfractionation of Extract 37 (SA_L_1) by Analytical-Scale High Performance Liquid Chromatography (HPLC)

A part (100 mg) of the active extract 37 (leaf extract from SA, accession 1, code SA_L_1, Table A3) was pre-treated with SPE Strata® C18-U cartridges (55 µm, 70 Å, 100 mg, 1 mL) (Phenomenex, Torrance, CA, USA). Analytical-scale HPLC fractionation of the SPE-treated extract was performed with an UFLC Prominence system (Shimadzu, Columbia, MD, USA) consisting of a pump (LC-20AD), a column oven (CTO-20AC), a UV/Vis detector (SPD-20AV), an autosampler (SIL-10AP), a fraction collector (FRC-10A) and a Phenomenex C18 Synergi HydroRP 80A column (150 × 4.6 mm i.d., 4 μm, 100 Å) (Phenomenex, Torrance, CA, USA). A flow rate of 0.7 mL/min was used with a combination of solvent A (1% formic acid in ACN) and solvent B (1% formic acid in H_2_O). A gradient elution method was used: (0–1 min 0% B, 1-5 min 0% to 20% B, 14–23 min 100% B, and 25–30 min 0% B). 5 μL of extract solution (15 mg/mL) was injected for each HPLC analysis. Therefore, the optimized separation method (50 min) of extract 37 was performed to fractionate SPE extract by time-slicing the HPLC elute from 5 to 47 min in microfractions of 0.25 min. The gradient elution method was optimized as follows: (0–1 min 0% B, 1–23 min 0% to 50% B, 23–28 min 50%, 28–38 min 50% to 100% B, 38–43 min 100% B, 43–46 min 100% to 0% B). We independently collected 168 microfractions into 3.5 mL test tubes. The separation/collection procedure was repeated three times. Once the microfractionation was finished, tubes were dried overnight at 35 °C using a Multivapor^TM^ P-12 vacuum parallel evaporator (Büchi Labortechnik AG, Flawil, Switzerland). PDB medium with 2% DMSO (100 μL) was added to each dried microfraction. They were shaken for 1 h to dissolve the entire content and a *F. oxysporum* conidia suspension (1 × 10^6^ conidia/mL) in PDB (100 μL) was subsequently added to each tube, following the procedure described previously in Section 3.4. Inhibition percentage of each treated conidia was calculated using the OD value of a control (100% viability) and related to the OD of each treatment. A high-resolution biochromatogram was then assembled by representing conidia germination inhibition percentages against chromatographic profile.

### 3.7. Purification and Identification of Most-Active Compounds from Extract 37 by Semipreparative-Scale HPLC

Extract 37 (400 mg) was also pre-treated with SPE Strata® C18-U cartridges (55 µm, 70 Å, 500 mg, 6 mL) (Phenomenex, Torrance, CA, USA). Most active microfractions were then collected using an UFLC Prominence system (Shimadzu, Columbia, MD, USA), operated in semipreparative mode, consisting of a pump (LC-20AD), a column oven (CTO-20AC), a UV/Vis detector (SPD-20AV), an autosampler (SIL-10AP), a fraction collector (FRC-10A) and equipped with a reversed-phase Phenomenex Luna C18 column (250 × 10 mm, 5 μm) (Phenomenex, Torrance, CA, USA) at 20 °C. Eight consecutive injections of SPE extract (500 μL per injection, 80 mg/mL in MeOH) were separated at a flow rate of 3 mL/min using solvents A (1% formic acid in ACN) and B (1% formic acid in H_2_O) and a gradient elution method: (0–1 min 0% B, 1–23 min 0% to 50% B, 23–28 min 50%, 28–38 min 50% to 100% B, 38–43 min 100% B, 43–46 min 100% to 0% B). The targeted peaks (i.e., fractions) were collected at retention time 14.0–14.1 min (2.2 mg), 19.3–19.5 min (1.3 mg), 20.5 min (1.9 mg), 25.3 min (2.0 mg), and 28.7–28.9 min (2.6 mg), to afford pure compounds **1**–**5**, respectively. Structures of isolated compounds were elucidated by ^1^H and ^13^C NMR on an Agilent DD2 600 MHz spectrometer (Bruker, Billerica, MA, USA) using CDCl_3_ as solvent. All shifts are given in δ (ppm) using tetramethylsilane (TMS) as internal standard. Coupling constants (J) in Hz. NMR data of compounds **1**–**5** are presented in Table A5. 

### 3.8. Statistical Analyses

TLiC and antifungal activity data were subjected to the Shapiro–Wilks test, parametric analysis of variance (ANOVA), and Tukey’s test (*p* < 0.05) using R project software version 3.0.2 (R Foundation, Vienna, Austria). In addition, the LC-MS-derived raw data files were pre-processed with MzMine 2.2 (Whitehead Institute for Biomedical Research, MA, USA) for feature (peak) detection, deconvolution, filtering, deisotopization, gap-filling, gap-filled, alignment, and normalization [28]. The list of detected features and inhibition percentages against *F. oxysporum* conidia were used to build the input dataset. The resulting matrix was used for multivariate statistics through sparse partial least squares discrimant analysis (sPLS-DA) using MetaboAnalyst 4.0 (McGill Univeristy, Quebec, Canada) [46] and single-*Y* orthogonal partial least squares (OPLS) regression using the SIMCA 13.0 software (Umetrics Inc., Umeå, Sweden).

## 4. Conclusions

Extracts of *A. indica* accessions from six different locations on the Colombian Caribbean coast exhibited variation in limonoid contents and antifungal data, ranging from 4.5 to 48.5 mg limonin/g dry extract and 0.08–44.8 μg/mL, respectively. However, a clear trend was not observed between climatic conditions and this data and no correlation was found between TLiC and IC_50_ values. The variation of LC-MS chemical profiles was also demonstrated, and the discrimination between locations per plant part was achieved through sPLS-DA models, indicating some particular metabolites (i.e., limonoids, flavonoids, and terpenes) as the most influencing features for the observed clustering. However, climatic conditions were not enough to rationalize such variations, so other biotic/abiotic factors should be explored in further studies. On the other hand, the biochemometrics-based analysis, combining LC-MS profiles with the antifungal data, led to the identification of bioactive azadirone-like limonoids avoiding several bioactivity-guided isolation steps during antifungal discovery. Thus, a single-Y OPLS-based model exposed five features, from a set of 84 extracts, which were related with the observed conidia inhibition. The high-resolution *F. oxysporum* conidia inhibition profiling in combination with RP-HPLC-ESI-MS data resulted in the identification of the five azadirone-like limonoids, of which, three of them were potent inhibitors of germination of *F. oxysporum* conidia. Nimonol **1** was the most active limonoid (IC_50_ = 1.48 ± 0.11 µM). This is the first report for these limonoids as *F. oxysporum* inhibitors, and consequently offers a rationale for the discovery of natural antifungals. Results then suggest that identified compounds are chemical agents that could be involved in further assays on *F. oxysporum* control.

## Figures and Tables

**Table 1:** Description of the *A. indica* samples and collecting locations within the Colombian Caribbean coast.

Location	Abb *^a^*	Colombian Caribbean Areas	A (m) *^b^*	Climate conditions			Samples	
				KGC *^c^*	T (˚C) *^d^*	RF (mm) *^e^*	*N ^f^*	PP *^g^*
Riohacha	R	La Guajira Peninsula	5	Bsh	28.3	588	9	L,F,S
San Pedro de la Sierra	SPS	The Sierra Nevada	1378	Am	18.9	2721	5	L,F,S
Santa Ana	SA	Magdalena River	25	Aw	24.0	1468	6	L,F,S
Santa Marta	SM	The Sierra Nevada	9	Bsh	28.3	512	5	L
Mompox	M	Magdalena River	16	Aw	27.5	1573	10	L
Montería	Mo	Sinú Belt	20	Aw	27.4	1225	5	L,F,S

*^a^* Abb = abbreviation; *^b^* A = Altitude (meter above mean sea level); *^c^* KGC = Köppen–Geiger climate classification system (BsH = hot semi-arid; Am = tropical monsoon; Aw = tropical wet (savanna); *^d^* T = Temperature (annual average, ˚C); *^e^* RF = rainfall (annual average, mm); *^f^* n = number of *A. indica* Accessions for each location; *^g^* PP = plant parts of *A. indica* employed for extraction (L = leaves; F = fruits; S = seeds) according to the natural offer of each sampled tree.

**Table 2:** Features (i.e., compounds) selected by OPLS model and annotated after LC-ESI-high-resolution MS (HRMS) analysis.

No *^a^*	*m/z* [M+H]^+^	t_R_ (min)	MF *^b^*	Annotation	Error
**1**	453.2649	14.1	C_28_H_37_O_6_^+^	hydroxyazadirone isomer 1	−1.76
**2**	469.2585	19.4	C_28_H_37_O_13_^+^	epoxyhydroxyazadirone	1.07
**3**	455.2791	20.5	C_28_H_39_O_10_^+^	dehydrohydroxyazadirone	1.32
**4**	485.2915	25.3	C_29_H_41_O_20_^+^	didehydrooxohydroxyazadirone	−2.47
**5**	453.2632	28.8	C_28_H_37_O_9_^+^	hydroxyazadirone isomer 2	1.99

*^a^* The numbering agrees the order of selected features in *S*-plot (Figure 5b); *^b^* MF = molecular formula of the quasi-molecular ion.

**Table 3:** Antifungal activity of limonoids **1**–**5** isolated from leaves of *A. indica*.

Compounds	1	2	3	4	5
**IC_50_*^a^***	1.48 ± 0.11	6.42 ± 0.26	15.17 ± 0.22	4.57 ± 0.23	10.07 ± 0.31

*^a^* Half maximal inhibitory concentration (IC_50_ in μM) for the inhibition of *F. oxysporum* conidia germination. All values are expressed as mean ± standard deviation (*n* = 4).

## Appendix A

**Table A1:** Coordinates for accessions of *A. indica* from Caribbean coast locations. Colors according to locations (Figure 2).

Location	Accession	Coordinates
San Pedro de la Sierra (SPS)	1	10°53′6.31″ N 74° 6′28.44″ W
	2	10°53′40.66″ N 74° 5′56.10″ W
	3	10°53′41.15″ N 74° 5′53.93″ W
	4	10°53′20.87″ N 74° 6′16.43″ W
	5	10°52′53.63″ N 74° 9′9.93″ W
Santa Ana (SA)	1	9°19′19.10″ N 74°34′46.31″ W
	2	9°19′39.14″ N 74°34′48.64″ W
	3	9°19′39.49″ N 74°34′47.71″ W
	4	9°19′39.62″ N 74°34′47.48″ W
	5	9°19′39.71″ N 74°34′47.29″ W
	6	9°19′13.63″ N 74°34′23.03″ W
Riohacha (R)	1	11°31′28.03″ N 72°54′7.55″ W
	2	11°31′27.97″ N 72°54′7.90″ W
	3	11°31′28.84″ N 72°54′8.95″ W
	4	11°31′30.62″ N 72°54′13.34″ W
	5	11°31′30.18″ N 72°54′13.74″ W
	6	11°31′29.21″ N 72°54′15.56″ W
	7	11°31′29.97″ N 72°54′16.63″ W
	8	11°31′29.68″ N 72°54′18.33″ W
	9	11°31′26.78″ N 72°54′11.65″ W
Mompox (M)	1	9°14′36.17″ N 74°25′37.73″ W
	2	9°14′36.06″ N 74°25′37.68″ W
	3	9°14′28.10″ N 74°25′22.49″ W
	4	9°14′29.11″ N 74°25′24.05″ W
	5	9°14′17.76″ N 74°25′15.06″ W
Santa Marta (SM)	1	11°13′26.45″ N 74°11′6.14″ W
	2	11°13′26.29″ N 74°11′6.63″ W
	3	11°13′26.16″ N 74°11′5.90″ W
	4	11°13′26.07″ N 74°11′6.15″ W
	5	11°13′25.92″ N 74°11′6.57″ W
Montería (Mo)	1	8°45′44.71″ N 75°52′39.16″ W
	2	8°45′50.04″ N 75°52′43.17″ W
	3	8°45′50.12″ N 75°52′43.08″ W
	4	8°45′50.20″ N 75°52′43.00″ W
	5	8°45′50.27″ N 75°52′42.96″ W
	6	8°45′5.16″ N 75°53′27.34″ W
	7	8°45′5.34″ N 75°53′27.24″ W
	8	8°45′3.99″ N 75°53′27.19″ W
	9	8°45′3.98″ N 75°53′26.99″ W
	10	8°45′6.67″ N 75°53′28.20″ W

**Table A2:** Total limonoid content (TLiC) of eighty-four *A. indica*-derived crude extracts.

No	Extract *^a^*	TLiC *^b^*	No	Extract *^a^*	TLiC *^b^*	No	Extract *^a^*	TLiC *^b^*	No	Extract *^a^*	TLiC *^b^*
1	SPS_L_1	13.5 ± 0.5	22	R_F_4	25.7 ± 1.5	43	SA_F_2	17.6 ± 2.9	64	Mo_L_9	12.4 ± 0.7
2	SPS_L_2	14.8 ± 0.8	23	R_F_5	21.1 ± 2.1	44	SA_F_4	15.1 ± 1.8	65	Mo_L_10	11.4 ± 0.8
3	SPS_L_3	16.0 ± 0.6	24	R_F_6	25.5 ± 1.5	45	SA_F_5	13.1 ± 1.5	66	Mo_F_1	13.7 ± 3.8
4	SPS_L_4	15.8 ± 0.3	25	R_F_7	28.8 ± 2.4	46	SA_F_6	17.0 ± 2.7	67	Mo_F_2	15.6 ± 0.7
5	SPS_L_5	15.0 ± 1.0	26	R_F_8	21.3 ± 2.1	47	SA_S_2	48.5 ± 1.7	68	Mo_F_3	16.2 ± 0.9
6	SPS_F_4	20.4 ± 0.3	27	R_F_9	23 ± 1.1.8	48	SA_S_4	24.9 ± 4.2	69	Mo_F_4	15.2 ± 0.4
7	SPS_F_5	20.6 ± 1.4	28	R_S_1	42.8 ± 3.2	49	SA_S_5	38.5 ± 9.4	70	Mo_F_5	15.7 ± 0.4
8	SPS_S_4	23.0 ± 0.4	29	R_S_2	33.4 ± 1.3	50	SA_S_6	42.5 ± 5.8	71	Mo_F_6	15.1 ± 1.1
9	SPS_S_5	24.3 ± 0.7	30	R_S_3	43.6 ± 1.0	51	M_L_1	19.2 ± 0.7	72	Mo_F_7	17.6 ± 2.9
10	R_L_1	17.9 ± 1.6	31	R_S_4	34.2 ± 1.5	52	M_L_2	10.5 ± 0.8	73	Mo_S_1	13.9 ± 3.0
11	R_L_2	14.7 ± 3.0	32	R_S_5	34.7 ± 6.1	53	M_L_3	12.2 ± 0.8	74	Mo_S_2	17.9 ± 4.2
12	R_L_3	13.2 ± 2.6	33	R_S_6	31.3 ± 2.2	54	M_L_4	13.1 ± 1.5	75	Mo_S_3	16.1 ± 1.3
13	R_L_4	14.4 ± 2.1	34	R_S_7	42.4 ± 2.4	55	M_L_5	14.6 ± 7.3	76	Mo_S_4	15.5 ± 1.3
14	R_L_5	17.1 ± 1.4	35	R_S_8	40.6 ± 2.7	56	Mo_L_1	13.7 ± 1.8	77	Mo_S_5	14.6 ± 4.4
15	R_L_6	14.8 ± 1.8	36	R_S_9	43.9 ± 1.9	57	Mo_L_2	13.4 ± 2.4	78	Mo_S_6	19.6 ± 1.8
16	R_L_7	13.7 ± 1.7	37	SA_L_1	6.7 ± 0.5	58	Mo_L_3	12.4 ± 1.0	79	Mo_S_7	16.5 ± 1.4
17	R_L_8	17.3 ± 0.5	38	SA_L_2	4.5 ± 0.4	59	Mo_L_4	15.0 ± 0.3	80	SM_L_1	32.6 ± 2.4
18	R_L_9	17.6 ± 0.7	39	SA_L_3	7.4 ± 0.6	60	Mo_L_5	12.5 ± 0.4	81	SM_L_2	33.0 ± 2.3
19	R_F_1	21.5 ± 1.0	40	SA_L_4	8.7 ± 0.8	61	Mo_L_6	13.1 ± 0.9	82	SM_L_3	31.7 ± 1.0
20	R_F_2	21.4 ± 1.2	41	SA_L_5	7.6 ± 0.8	62	Mo_L_7	8.2 ± 0.5	83	SM_L_4	43.6 ± 4.3
21	R_F_3	20.0 ± 1.1	42	SA_L_6	8.8 ± 0.5	63	Mo_L_8	12.9 ± 0.6	84	SM_L_5	38.7 ± 2.2

*^a^* Extracts obtained from the *A. indica* accessions from different locations (SPS = San Pedro de la Sierra; R = Riohacha; SA = Santa Ana; M = Mompox; Mo = Monteria; SM = Santa Marta) and plant parts (L = leaves; F = fruits; S = seeds); *^b^* Total Limonoid Content expressed as mg equivalent to limonin per g dry extract (mg limonin/g DE); *^c^* Half maximal inhibitory concentration (IC_50_ in μg/mL) for the inhibition of *F. oxysporum* conidia germination. All values are expressed as mean ± standard deviation (*n* = 4). Colors according to locations (Figure 2).

**Table A3:** Antifungal activity of eighty-four *A. indica*-derived crude extracts.

No	Extract *^a^*	IC_50_ *^c^*	No	Extract *^a^*	IC_50_ *^c^*	No	Extract *^a^*	IC_50_ *^c^*	No	Extract *^a^*	IC_50_ *^c^*
1	SPS_L_1	0.56 ± 0.04	22	R_F_4	9.63 ± 0.58	43	SA_F_2	3.53 ± 0.28	64	Mo_L_9	0.39 ± 0.05
2	SPS_L_2	1.56 ± 0.12	23	R_F_5	19.8 ± 0.99	44	SA_F_4	0.77 ± 0.03	65	Mo_L_10	0.16 ± 0.01
3	SPS_L_3	0.23 ± 0.02	24	R_F_6	0.65 ± 0.03	45	SA_F_5	0.13 ± 0.01	66	Mo_F_1	44.8 ± 1.79
4	SPS_L_4	0.12 ± 0.01	25	R_F_7	0.54 ± 0.04	46	SA_F_6	0.68 ± 0.05	67	Mo_F_2	4.91 ± 0.39
5	SPS_L_5	0.25 ± 0.03	26	R_F_8	0.35 ± 0.04	47	SA_S_2	2.78 ± 0.25	68	Mo_F_3	37.1 ± 3.71
6	SPS_F_4	2.72 ± 0.27	27	R_F_9	0.74 ± 0.03	48	SA_S_4	32.6 ± 3.26	69	Mo_F_4	2.60 ± 0.16
7	SPS_F_5	1.35 ± 0.08	28	R_S_1	0.25 ± 0.02	49	SA_S_5	6.44 ± 0.52	70	Mo_F_5	33.4 ± 4.01
8	SPS_S_4	0.28 ± 0.03	29	R_S_2	0.22 ± 0.01	50	SA_S_6	35.8 ± 2.15	71	Mo_F_6	0.88 ± 0.04
9	SPS_S_5	0.27 ± 0.03	30	R_S_3	0.18 ± 0.01	51	M_L_1	2.32 ± 0.09	72	Mo_F_7	1.09 ± 0.12
10	R_L_1	0.32 ± 0.02	31	R_S_4	0.11 ± 0.01	52	M_L_2	26.8 ± 2.14	73	Mo_S_1	1.95 ± 0.14
11	R_L_2	3.64 ± 0.44	32	R_S_5	7.35 ± 0.29	53	M_L_3	2.12 ± 0.08	74	Mo_S_2	1.63 ± 0.15
12	R_L_3	1.03 ± 0.10	33	R_S_6	0.08 ± 0.01	54	M_L_4	38.6 ± 2.32	75	Mo_S_3	1.77 ± 0.09
13	R_L_4	0.45 ± 0.05	34	R_S_7	0.55 ± 0.06	55	M_L_5	33.9 ± 3.39	76	Mo_S_4	29.8 ± 2.68
14	R_L_5	9.67 ± 0.77	35	R_S_8	0.21 ± 0.02	56	Mo_L_1	41.6 ± 3.74	77	Mo_S_5	3.67 ± 0.18
15	R_L_6	28.2 ± 2.5	36	R_S_9	0.78 ± 0.05	57	Mo_L_2	27.9 ± 1.40	78	Mo_S_6	35.6 ± 4.27
16	R_L_7	7.52 ± 0.83	37	SA_L_1	0.14 ± 0.01	58	Mo_L_3	3.12 ± 0.22	79	Mo_S_7	0.88 ± 0.04
17	R_L_8	0.41 ± 0.04	38	SA_L_2	0.17 ± 0.02	59	Mo_L_4	31.2 ± 3.74	80	SM_L_1	2.56 ± 0.23
18	R_L_9	0.53 ± 0.03	39	SA_L_3	1.25 ± 0.08	60	Mo_L_5	42.9 ± 5.15	81	SM_L_2	38.7 ± 4.26
19	R_F_1	18.8 ± 1.32	40	SA_L_4	0.98 ± 0.11	61	Mo_L_6	27.8 ± 1.95	82	SM_L_3	2.36 ± 0.21
20	R_F_2	3.37 ± 0.40	41	SA_L_5	21.6 ± 1.30	62	Mo_L_7	1.46 ± 0.13	83	SM_L_4	1.25 ± 0.10
21	R_F_3	8.74 ± 1.05	42	SA_L_6	4.51 ± 0.18	63	Mo_L_8	0.69 ± 0.08	84	SM_L_5	3.54 ± 0.28

*^a^* Extracts obtained from the *A. indica* accessions from different locations (SPS = San Pedro de la Sierra; R = Riohacha; SA = Santa Ana; M = Mompox; Mo = Monteria; SM = Santa Marta) and plant parts (L = leaves; F = fruits; S = seeds); *^b^* Total Limonoid Content expressed as mg equivalent to limonin per g dry extract (mg limonin/g DE); *^c^* Half maximal inhibitory concentration (IC_50_ in μg/mL) for the inhibition of *F. oxysporum* conidia germination. All values are expressed as mean ± standard deviation (*n* = 4). Colors according to locations (Figure 2).

**Table A4:** Features (i.e., compounds) selected by sPLS-DA and annotated (identification at level 3 [29]) after LC-ESI-HRMS analysis.

Leaves						Fruits						Seeds					
No *^a^*	*m/z* [M+H]^+^	t_R_ (min)	MF *^b^*	Annotation	Error	No *^a^*	*m/z* [M+H]^+^	t_R_ (min)	MF *^b^*	Annotation	Error	No *^a^*	*m/z* [M+H]^+^	t_R_ (min)	MF *^b^*	Annotation	Error
1	287.0563	18.2	C_15_H_11_O_6_^+^	luteolin	−2.79	1	249.1133	11.8	C_14_H_17_O_4_^+^	hydroxyfraxinellone	−2.79	1	587.2479	27.6	C_31_H_39_O_11_^+^	humilinolide D	2.21
2	635.2712	18.9	C_32_H_43_O_13_^+^	azadirachtin M	−1.42	2	261.1137	8.1	C_15_H_17_O_4_^+^	azedararide	−1.42	2	527.3379	23.7	C_32_H_47_O_6_^+^	azadirachtol	−1.33
3	613.3021	13.7	C_34_H_45_O_10_^+^	ohchinolal	−1.47	3	233.1168	9.9	C_14_H_17_O_3_^+^	fraxinellone	−1.47	3	721.2716	29.5	C_35_H_45_O_16_^+^	azadirachtin A	−1.25
4	995.4837	18.5	C_50_H_75_O_20_^+^	NA*^c^*	1.41	4	357.1325	16.4	C_20_H_21_O_6_^+^	flowerone	1.41	4	523.3041	20.9	C_32_H_43_O_6_^+^	azadironol	3.44
5	571.2919	19.9	C_32_H_43_O_9_^+^	swietenin E	−2.10	5	345.0986	10.4	C_18_H_17_O_7_^+^	eupatilin	−2.10	5	555.2963	17.6	C_32_H_43_O_8_^+^	meliacinol	−1.08
6	239.1081	14.5	C_16_H_15_O_2_^+^	methoxychalcone	−3.76	6	223.2069	21.9	C_15_H_27_O^+^	α-cadinol	−3.76	6	635.2711	18.9	C_32_H_43_O_13_^+^	azadirachtin M	−1.26
7	597.3069	24.8	C_34_H_45_O_9_^+^	salannin	−1.00	7	291.0851	5.81	C_15_H_15_O_6_^+^	catechin	−1.00	7	369.2049	26.1	C_23_H_29_O_4_^+^	nimbothalin	4.33
8	999.5148	22.5	C_50_H_79_O_20_^+^	NA*^c^*	1.60	8	557.2766	34.1	C_31_H_41_O_9_^+^	swietenin C	1.60	8	499.3069	26.9	C_30_H_43_O_6_^+^	azadirolic acid	−2.00
9	223.2071	21.9	C_15_H_27_O^+^	α-cadinol	−4.48	9	527.3361	23.7	C_32_H_47_O_6_^+^	azadirachtol	−4.48	9	467.2421	34.8	C_28_H_35_O_6_^+^	azadiradionolide	2.57
10	999.5171	21.3	C_50_H_79_O_20_^+^	NA*^c^*	−0.70	10	555.2971	17.6	C_32_H_43_O_8_^+^	meliacinol	−0.70	10	513.2859	37.4	C_30_H_41_O_7_^+^	salimuzzalin	−1.36

*^a^* The numbering agrees the order of ranked features in loadings plots, which are exposed in Figure 4d–f; *^b^* MF = molecular formula of the quasi-molecular ion; *^c^* NA = not annotated.

**Table A5:** ^1^H (400 MHz, CDCl_3_) and ^13^C (100 MHz, CDCl_3_) NMR data of identified compounds **1**–**5**.

	1			2			3			4			5		
No	Type	*δ*_C_	*δ*_H_, mult, *J* (Hz)	Type	*δ*_C_	*δ*_H_, mult, *J* (Hz)	Type	*δ*_C_	*δ*_H_, mult, *J* (Hz)	Type	*δ*_C_	*δ*_H_, mult, *J* (Hz)	Type	*δ*_C_	*δ*_H_, mult, *J* (Hz)
C-1	CH	157.1	7.14, d, 10.2	CH	157.4	7.10, d, 9.8	CH_2_	33.5	1.41–1.79, m	CH	157.3	7.05, d, 10.2	CH	157.9	7.12, d, 10.3
C-2	CH	125.9	5.92, d, 10.2	CH	126.1	5.95, d, 9.8	CH_2_	38.3	2.18-2.41, m	CH	126.8	5.89, d, 10.2	CH	126.3	5.93, d, 10.3
C-3	C	206.1		C	205.9		C	209.7		C	204.1		C	205.7	
C-4	C	40.4		C	40.5		C	41.2		C	43.4		C	40.8	
C-5	CH	49.8	2.20, d, 11.5	CH	50.2	2.23, d, 11.8	CH	47.1	2.07, d, 11.0	CH	48.6	2.13, d, 11.31	CH	49.6	2.76, d, 12.2
C-6	CH	68.2	4.39, dd, 11.5, 2.4	CH	68.6	4.34, dd, 11.8, 2.5	CH	68.8	4.17, dd, 11.0, 3.1	CH	67.5	4.25, dd, 2.7, 11.1	CH	76.4	5.44, dd, 12.2, 2.6
C-7	CH	79.0	5.34, d, 2.4	CH	77.5	4.95, d, 2.7	CH	79.2	5.24, d, 3.1	CH	82.3	5.29, d, 2.7	CH	68.4	4.06, d, 2.6
C-8	C	45.4		C	42.7		C	43.5		C	45.6		C	45.2	
C-9	CH	37.3	2.15–2.28, m	CH	39.3	2.50–2.67, m	CH	34.3	2.60–2.75, m	CH	38.6	2.22, dd, 14.1, 5.1	CH	37.3	2.31, d, 12.6, 6.4
C-10	C	43.4		C	41.7		C	38.5		C	42.1		C	43.0	
C-11	CH_2_	16.6	1.95–2.04, m (Ha) 1.70–1.81, m (Hb)	CH_2_	16.4	1.90–2.02 m (Ha) 1.71–1.87, m (Hb)	CH_2_	16.5	1.40–1.89, m	CH_2_	16.5	2.35–2.50, m (Ha) 2.05–2.21, m (Hb)	CH_2_	16.3	2.21–2.38, m (Ha) 2.02–2.29, m (Hb)
C-12	CH_2_	32.5	1.84–1.92, m (Ha) 2.26–2.33, m (Hb)	CH_2_	29.1	1.71–1.87, m (Ha) 2.40–2.42, m (Hb)	CH_2_	31.7	1.44–1.89, m	CH_2_	32.9	2.38–2.48, m (Ha) 2.79–2.85, m (Hb)	CH_2_	33.5	2.02–2.29, m (Ha) 2.36–2.43, m (Hb)
C-13	C	47.3		C	45.5		C	47.1		C	59.5		C	46.9	
C-14	C	158.6		C	73.0		C	158.6		C	193.4		C	158.5	
C-15	CH	119.4	5.45, dd, 2.7, 1.9	CH	57.3	3.48–3.55, m	CH	119.7	5.35, m	CH	123.9	5.75, s	CH	119.0	5.56, dd, 3.5, 1.7
C-16	CH_2_	34.2	1.68–1.75, m (Ha) 2.32–2.41, m (Hb)	CH_2_	32.2	1.59–1.65, m (Ha) 2.10–2.31, m (Hb)	CH_2_	33.1	1.44–1.89, m	C	208.0		CH_2_	34.5	1.95–2.01, m (Ha) 2.41, ddd, 14.4, 7.2, 3.5 (Hb)
C-17	CH	51.8	2.84, dd, 10.6, 7.1	CH	39.4	2.66, dd, 3.6, 2.9	CH	51.4		CH	56.4	3.20, dd, 8.1, 1.7	CH	51.6	2.44, dd, 7.2, 3.5
C-18	CH_3_	20.4	0.85, s	CH_3_	21.8	0.95, s	CH_3_	20.7	0.77, s	CH_3_	18.4	1.08, s	CH_3_	20.6	0.84, s
C-19	CH_3_	20.1	1.15, s	CH_3_	20.4	1.11, s	CH_3_	16.6	0.81, s	CH_3_	16.1	1.17, s	CH_3_	14.0	1.14, s
C-20	C	124.3		C	123.8		C	124.5		CH	143.6	3.33, m	C	124.5	
C-21	CH	142.8	7.25, dd, 1.7, 1.0	CH	142.8	7.36, dd, 1.6, 0.8	CH	142.6	7.35, m	C	203.1	9.78, d, 7.5	CH	142.6	7.26, dd, 1.7, 0.8
C-22	CH	111.1	6.32, dd, 1.7, 1.0	CH	111.3	6.19, dd, 1.6, 0.8	CH	111.7	6.17, m	CH_2_	32.1	2.02-2.34, m	CH	111.2	6.25, dd, 1.7, 0.8
C-23	CH	139.4	7.38, t, 1.7	CH	139.6	7.16, t, 1.6	CH	139.5	7.19, m	CH_3_	21.9	1.24, t, 8.2	CH	139.4	7.37, t, 1.7
C-28	CH_3_	31.6	1.27, s	CH_3_	31.8	1.19, s	CH_3_	31.6	1.132, s	CH_3_	25.0	1.22, s	CH_3_	26.9	1.19, s
C-29	CH_3_	20.6	1.30, s	CH_3_	19.4	1.32, s	CH_3_	19.5	1.21, s	CH_3_	21.3	1.25, s	CH_3_	19.5	1.25, s
C-30	CH_3_	26.8	1.39, s	CH_3_	21.8	1.39, s	CH_3_	26.2	1.25, s	CH_3_	25.7	1.26, s	CH_3_	20.1	1.32, s
COCH_3_	C	171.8		C	171.6		C	172.2		C	169.2		C	172.3	
COCH_3_	CH_3_	21.1	2.05, s	CH_3_	21.4	2.13, s	CH_3_	21.5	2.01, s	CH_3_	19.7	1.95, s	CH_3_	21.5	2.00, s
OCH_3_	-			-			-			CH_3_		3.50, s	-		
